# Comprehensive transcriptome analysis of erythroid differentiation potential of olive leaf in haematopoietic stem cells

**DOI:** 10.1111/jcmm.16752

**Published:** 2021-06-27

**Authors:** Shinji Kondo, Farhana Ferdousi, Ken Yamauchi, Sofya Suidasari, Miki Yokozawa, Mohamed Moncef Harrabi, Ken‐ichi Tominaga, Hiroko Isoda

**Affiliations:** ^1^ R&D Center for Tailor‐Made QOL University of Tsukuba Tsukuba Japan; ^2^ Alliance for Research on the Mediterranean and North Africa (ARENA) University of Tsukuba Tsukuba Japan; ^3^ AIST‐University of Tsukuba Open innovation laboratory for food and medicinal resource engineering (FoodMed‐OIL) University of Tsukuba Tsukuba Japan; ^4^ Faculty of Life and Environmental Sciences University of Tsukuba Tsukuba Japan; ^5^ Nutrition Act Co., Ltd. Tokyo Japan

**Keywords:** anaemia, DNA microarray, erythropoiesis, haematopoietic stem cell, iron homeostasis, olive leaf

## Abstract

Anaemia is one of the leading causes of disability in young adults and is associated with increased morbidity and mortality in elderly. With a global target to reduce the disease burden of anaemia, recent researches focus on novel compounds with the ability to induce erythropoiesis and regulate iron homeostasis. We aimed to explore the biological events and potential polypharmacological effects of water‐extracted olive leaf (WOL) on human bone marrow–derived haematopoietic stem cells (hHSCs) using a comprehensive gene expression analysis. HPLC analysis identifies six bioactive polyphenols in the WOL. Treatment with WOL for 12 days regulated gene expressions related to erythroid differentiation, oxygen homeostasis, iron homeostasis, haem metabolism and Hb biosynthesis in hHSCs. Functional clustering analysis reveals several major functions of WOL such as ribosomal biogenesis and mitochondrial translation machinery, glycolytic process, ATP biosynthesis and immune response. Additionally, the colonies of both primitive and mature erythroid progenitors, CFU‐E and BFU‐E, were significantly increased in WOL‐treated hHSCs. The expressions of erythroid markers, CD47, glycophorin A (GYPA), and transferrin receptor (TFRC) and adult Hb subunits‐HBA and HBB were also confirmed in immunofluorescent staining and flow cytometer analysis in WOL‐treated hHSCs. It is well known that induction of lineage‐specific differentiation, as well as the maturation of early haematopoietic precursors into fully mature erythrocytes, involves multiple simultaneous biological events and complex signalling networks. In this regard, our genome‐wide transcriptome profiling with microarray study on WOL‐treated hHSCs provides general insights into the multitarget prophylactic and/or therapeutic potential of WOL in anaemia and other haematological disorders.

## 1 INTRODUCTION

Anaemia is a global public health problem affecting one‐third population worldwide. According to the World Health Organization (WHO), children under 5 years old, pregnant women and people older than 60 years are particularly at risk of developing anaemia with a worldwide prevalence of 47∙4%, 41∙8% and 23∙9%, respectively. Iron deficiency is estimated to cause half of all causes of anaemia. Other causes include physiological blood loss in women, acute and chronic infections, micronutrient deficiencies other than iron, and haemoglobinopathies. In adolescents and young adults (15‐59 years of age), iron‐deficiency anaemia is one of the leading causes of disability, causing extreme fatigue, decreased immunity, reduced work capacity, dizziness, headaches, hair loss, hearing deficit, anxiety and depression. In elderly, presence of anaemia is associated with worse prognosis of any disease, increased morbidity and mortality, decline in cognitive function, dementia and increased fracture risk. Current available treatment options for anaemia include iron or other nutrient supplementation, nutritional interventions in the form of food fortification, blood transfusion, erythropoietic agent infusion and anti‐inflammatory therapies.^1^, ^2^, ^3^, ^4^, ^5^ However, conventional treatments are not without adverse effects. Iron supplementation can cause abdominal cramping, nausea, constipation and dark stools. Blood transfusion may cause allergic, immune and febrile reactions and may increase the risk of acquiring blood‐borne diseases. Food fortification is not an accessible option for low‐ and middle‐income countries. On the other hand, there is a growing demand for innovative plant‐based nutritional alternatives for vegans and vegetarians who are more susceptible to anaemia.^6^, ^7^ In this context, medicinal plants with bioactive components have been receiving considerable attention from researchers as natural hematinic agents complemented with or alternatives to conventional treatment.^8^

Haematopoietic stem cells (HSCs) are the primary multipotent stem cells that can self‐renew as well as can further differentiate into a hierarchy of committed progenitors that ultimately give rise to mature blood cells through specific regulation of signal molecules.^9^ Multicomponent plant extracts may target multiple biological events to alter and/or control cell lineage differentiation of HSCs.^10^ Understanding the molecular functions and biological processes triggered by plant extracts will provide more general insights into their effects on stem cell regulation as well as facilitate their therapeutic use targeting HSCs.

In this regard, olive leaf extract (OLE) contains a wide variety of bioactive polyphenols that work synergistically and exert polypharmacological effects.^11^ We have previously reported that OLE induces monocyte/macrophage differentiation^12^ whereas its components apigetrin^13^ and apigenin^14^ induce erythroid differentiation in human chronic myelogenous leukaemia cell line K562. Additionally, phenolic compounds of OLE luteolin‐7‐glucoside and apigenin‐7‐glucoside could direct HSC differentiation towards erythroid lineage.^15^ However, the effects of multicomponent OLE on HSCs have not been evaluated yet.

In the present study, we have performed a comprehensive gene expression analysis of the regulatory networks in WOL‐treated human HSCs (hHSCs) to develop a new understanding of WOL's mechanism of action. We have also evaluated the effect of WOL treatment on the proliferation and differentiation pattern of hHSCs using colony forming cell (CFC) assay. Additionally, protein expression of several erythroid markers and haemoglobin (Hb) subunits was investigated. Our study presents a detailed analysis of biological events in hHSCs induced by WOL and highlights its multilevel exploitable targets for anaemia therapy.

## 2 MATERIALS AND METHODS

Detailed methodology is described in Appendix S1.

### 2.1 Extraction of olive leaves

Fresh olive leaves were harvested from a farm in Siliana Province at the central west of Tunisia in the middle of December 2018 and were shed‐dried at room temperature (RT) between 20 and 25°C.

### 2.2 Cell culture and sample treatment

The human chronic leukaemia cell line K562 was obtained from the Riken Cell Bank (RCB0027). Human bone marrow CD34+ progenitor cells were purchased from Lonza Inc (Lonza).

### 2.3 RNA extraction

Isogen reagent (311‐02501, Nippon Gene) was used to extract total RNA from K562 cells after treatment for 6 days and from hHSCs on day 9 (D0 before treatment) and day 21 (D12 after treatment), following the manufacturer's instructions. Concentrations of total RNA were quantified with NanoDrop 2000 spectrophotometer (Thermo Scientific).

### 2.4 DNA microarray analysis

DNA microarray was performed on GeneAtlas™ System using GeneChip™ 3′ IVT PLUS Reagent Kit and GeneAtlas™ Hybridization, Wash, and Stain Kit for 3′ IVT Arrays (Applied Biosystems, Thermo Fisher Scientific Inc), following the manufacturer's instructions. Gene annotation and pathway analysis were conducted using an online data mining tool DAVID ver. 6.8 and the Molecular Signatures Database (MSigDB) ver. 7.1 of the Gene Set Enrichment Analysis (GSEA) software.^16^ Heat maps were generated using visualization software Morpheus (https://software.broadinstitute.org/morpheus).

### 2.5 Statistical analysis

Statistical analyses were performed with GraphPad Prism version 8.0 (GraphPad Software, Inc). Data were represented as the mean ± standard error of the mean (SEM). An unpaired two‐tailed Student's *t* test was used to compare between two groups. A one‐way ANOVA followed by Dunnett's post hoc test was performed to compare the treatment groups to a control group. A *P*‐value <.05 was considered as significant.

## 3 RESULTS

### 3.1 Bioactive compounds in WOL

To identify and quantify the components in WOL, HPLC analysis was carried out. Table 1 shows the average amount of apigenin, apigenin‐7‐O‐glucoside, hydroxytyrosol, luteolin, luteolin‐7‐O‐glucoside and oleuropein (mg/g of dry weight) in OLE. In descending order of concentration, oleuropein was the main component with the concentration of 159.250 ± 0.396 mg/g dry weight and followed by luteolin‐7‐O‐glucoside (30.590 ± 0.129 mg/g). Other identified polyphenols were hydroxytyrosol, apigenin‐7‐O‐glucoside, luteolin and apigenin.

**TABLE 1:** Average amounts of the compounds in the water extract of olive leaf (WOL) by detection using HPLC

Compound	Amount (mg/g dry weight)
Apigenin	0.711 ± 0.003
Apigenin‐7‐O‐glucoside	1.763 ± 0.030
Hydroxytyrosol	3.569 ± 0.013
Luteolin	1.588 ± 0.008
Luteolin‐7‐O‐glucoside	30.590 ± 0.129
Oleuropein	159.250 ± 0.396

### 3.2 Confirmation of differentiation‐inducing effects of WOL‐treated K562 cells

Firstly, we confirmed the differentiation‐inducing potential of the WOL in K562 cells following our previous studies.^12^, ^14^ K562 is a leukaemic cell line but retains similar self‐renewal and pluripotent properties of HSCs and is commonly used as a cell model for studies of HSC differentiation.^17^ To evaluate the effect of WOL on K562 cell proliferation, 3‐(4,5‐dimethylthiazol‐2‐yl)‐2,5‐diphenyltetrazolium bromide (MTT) assay was performed. Figure S1A shows that cell proliferation was decreased to 66.1% at the concentration of 120 μg/mL WOL treatment and to 29.0% at 150 μg/mL in a dose‐dependent manner, compared to the untreated cells. To distinguish the cell death from the growth inhibition by WOL treatment, viable cells were counted using the Guava ViaCount flow cytometer. The cell viability was decreased to 71.0% at the concentration of 120 μg/mL compared to the untreated cells. Excessive cell death was observed at the concentration of 150 μg/mL (Figure S1B).

Figure S2A shows that the expressions of erythroid markers *CD36*, transferrin receptor (*TFRC*) and glycophorin A (*GYPA*) were significantly increased in the WOL‐treated K562 cells compared to the untreated control cells. WOL‐treated K562 cells also showed significant up‐regulation of the expressions of Hb subunit beta (*HBB*: 644.5%), Hb subunit alpha (*HBA*: 832.9%) and Hb subunit gamma (*HBG*: 368.6%) (Figure S2B). Analysis in fluorescence microscopy also indicated increased protein expressions of TFRC, GYPA and HBB in the WOL‐treated K562 cells compared to the control cells (Figure S3).

### 3.3 WOL induces erythroid marker *GYPA* expression in hHSC

**FIGURE 1:**
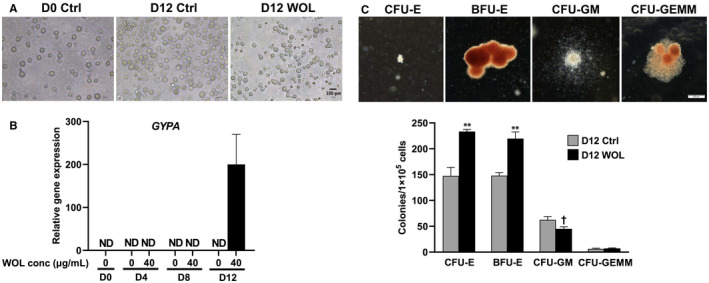
Effect of WOL on hHSC morphology and erythroid differentiation marker. A, The hHSCs were treated with 40 µg/mL WOL for 12 d and then observed under an optical microscope at a magnification of 200×. Scale bars represent 100 μm. B, Gene expressions of erythroid marker GYPA in WOL‐treated hHSCs were examined by real‐time PCR. The mRNA expressions were normalized to HPRT1 as internal control and represented as the mean ± SE for n = 4 in a 96‐well PCR plate. C, Morphology of colonies at 14‐d culture after WOL treatment was observed under an inverted microscope at a magnification of 40×. Scale bars represent 200 μm. Colonies were counted and classified according to their morphology. The data were represented as the mean ± SE for n = 3. ^†^ *P* < .1, ^**^ *P* < .01 by unpaired two‐tailed Student's *t* test

Figure 1A shows morphological changes in WOL‐treated hHSCs. The number and size of D12 control cells were increased compared to those of D0 control cells. On the other, the number of cells decreased, and the size of cells shrank by WOL treatment for 12 days.

Next, we examined the effect of WOL in the expression of glycophorin A (*GYPA*), the main marker of erythroid differentiation, to determine the optimum duration of WOL treatment for evaluation of biological events underlying its differentiation‐inducing effects. Figure 1B shows gene expression of GYPA in WOL‐treated hHSCs by real‐time PCR. WOL (40 µg/mL)‐treated cells and control cells were undetected during D0 to D8; meanwhile, GYPA could be detected only in WOL‐treated cells on D12. Therefore, for further gene expression analysis, we chose D12 WOL‐treated and untreated control hHSCs.

### 3.4 WOL treatment significantly increased the colonies of primitive and mature erythroid progenitors

We performed CFC assay to evaluate the effect of WOL treatment on the proliferation and differentiation pattern of hHSCs. We found that WOL treatment significantly increased the numbers of both colony forming unit (CFU)‐erythroid (CFU‐E) and burst forming unit‐erythroid (BFU‐E) in hHSCs cultured in a semisolid methylcellulose‐based medium. On the other hand, multilineage progenitors, namely CFU‐granulocyte, macrophage (CFU‐GM) and CFU‐granulocyte, erythrocyte, macrophage, megakaryocyte (CFU‐GEMM), were decreased in WOL‐treated hHSCs (Figure 1C).

### 3.5 Differentially expressed genes (DEGs) in WOL‐treated hHSCs

**FIGURE 2:**
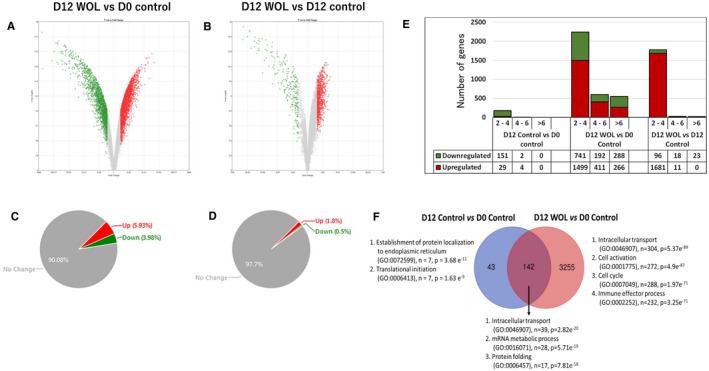
Filtering significantly differentially expressed genes (DEG) in WOL‐treated hHSCs. Volcano plot displaying DEGs between (A) WOL‐treated hHSCs on day 12 (D12 WOL) and untreated hHSCs on day 0 (D0 control), and (B) D12 WOL and untreated hHSCs on day 12 (D12 control). Volcano plots were generated using Transcriptome Analysis Console version 4 software. The vertical axis (*y*‐axis) corresponds to −log10 *P*‐value of the ANOVA *P*‐values, and the horizontal axis (*x*‐axis) displays linear fold change. The red and green dots represent the up‐regulated and down‐regulated genes, respectively. Pie chart displaying the percentages of up‐regulated and down‐regulated genes in DEGs of (C) D12 WOL *vs* D0 control, and (D) D12 WOL *vs* D12 control. (E) Distribution of fold changes (2‐4, 4‐6 and >6) of DEGs in different comparison conditions. (F) Venn diagram showing the unique and common number of DEGs in D12 control *vs* D0 control and D12 WOL *vs* D0 control. Top significantly enriched biological processes by each set of DEGs are shown

Next, we performed microarray analysis of WOL‐treated and untreated control hHSCs to explore the biological events that took place during the differentiation period. We prepared three technical replications of each treatment groups—day 0 control (D0 control), day 12 control (D12 control) and day 12 WOL‐treated group (D12 WOL), cultured from hHSCs purchased commercially from Lonza Inc Microarray was conducted on all three replicates of each treatment group. Genes satisfy both *P*‐value <.05 (one‐way between‐subjects ANOVA) and fold change (in linear space) >2.0 criteria simultaneously were considered as differentially expressed genes (DEGs). Figure 2A,B shows volcano plots displaying the DEGs between D12 WOL *vs* D0 control and D12 WOL *vs* D12 control, respectively. Pie charts show the percentages of significantly up‐ and down‐regulated genes in D12 WOL *vs* D0 control (Figure 2C), and in D12 WOL *vs* D12 control (Figure 2D). A total of 9.91% genes were differentially expressed between D12 WOL *vs* D0 control; among them, 5.93% were up‐regulated and 3.98% were down‐regulated. Between D12 WOL *vs* D12 control, 2.3% genes showed significantly different expressions; among them, 1.8% were up‐regulated genes and 0.5% were down‐regulated genes.

Figure 2E shows the distribution of fold changes of the DEGs in each comparison. When the cells were kept only in maintenance medium without any growth factors and other differentiation‐inducing factors, there were the least changes in gene expressions (D12 control *vs* D0 control). Only 186 genes showed more than 2‐fold change. On the other hand, hHSCs treated with WOL (D12 WOL) had 3397 DEGs when compared with untreated D0 control hHSCs; among them, 554 showed more than a 6‐fold change. Between D12 WOL *vs* D12 control, 1829 genes were differentially expressed mostly with 2‐ to 4‐fold change.

Next, we performed gene ontology (GO) analysis to compare the biological functions between WOL‐treated and untreated hHSCs (Figure 2F). D12 control hHSCs significantly enriched the GOs of the establishment of protein localization to the endoplasmic reticulum (GO:0072599) and translational initiation (GO:0006413), whereas both D12 control and D12 WOL commonly enriched intracellular transport (GO:0046907), mRNA metabolic process (GO:0016071) and protein folding (GO:0006457). WOL‐treated hHSCs significantly enriched intracellular transport (GO:0046907), cell activation (GO:0001775), cell cycle (GO:0007049) and immune effector process (GO:0002252).

### 3.6 Hallmark gene sets between D12 WOL *vs* D12 control hHSCs

**FIGURE 3:**
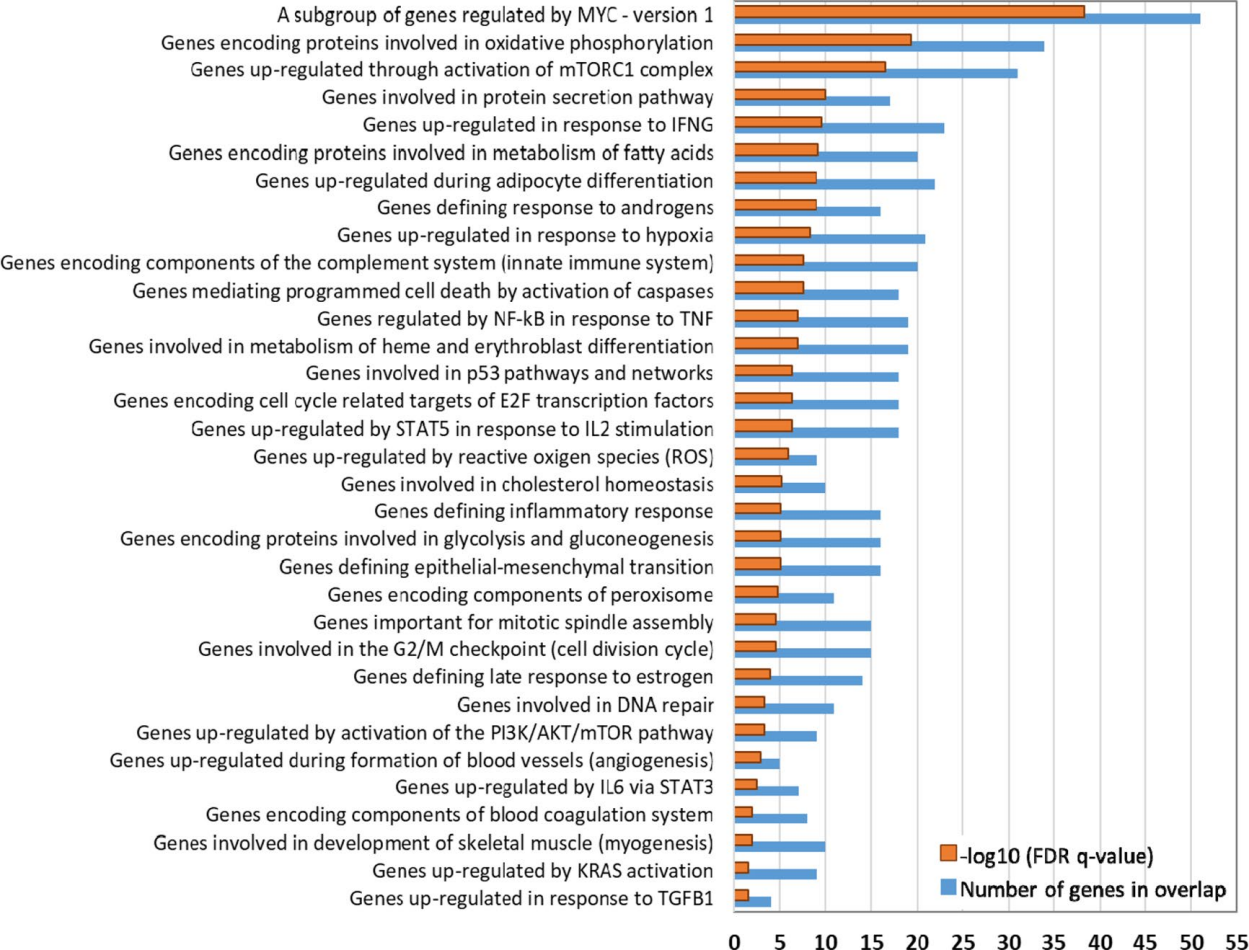
Hallmark gene sets between WOL‐treated and control hHSCs. Bar graph showing significantly enriched hallmark gene sets between D12 WOL and D12 control hHSCs. Hallmark gene sets were identified using the Molecular Signatures Database (MSigDB) of Gene Set Enrichment Analysis (GSEA) software (https://www.gsea‐msigdb.org/gsea/msigdb/index.jsp). Gene sets are arranged according to their significance measured as false discovery rate (FDR *q*‐value <0.05) after correcting for multiple hypothesis testing

We investigated significantly enriched hallmark gene sets between D12 WOL and D12 control hHSCs using the MSigDB of GSEA software. Hallmark gene sets represent specific well‐defined biological processes (BPs) and reduce noise and redundancy. There is an initial collection of 50 hallmarks, which collect and condense information from over 4,000 original overlapping gene sets.^18^ Figure 3 shows the significant gene sets enriched by the DEGs between D12 WOL and D12 control arranged in descending order of false discovery rate (FDR) q‐value. Several gene sets related to cell cycle, including cell division and cell growth, apoptosis, metabolism, autophagy, nutrient sensing and signalling and inflammatory response were significantly enriched by WOL treatment.

### 3.7 Gene functional clustering of BP ontologies between D12 WOL *vs* D12 control hHSCs

**FIGURE 4:**
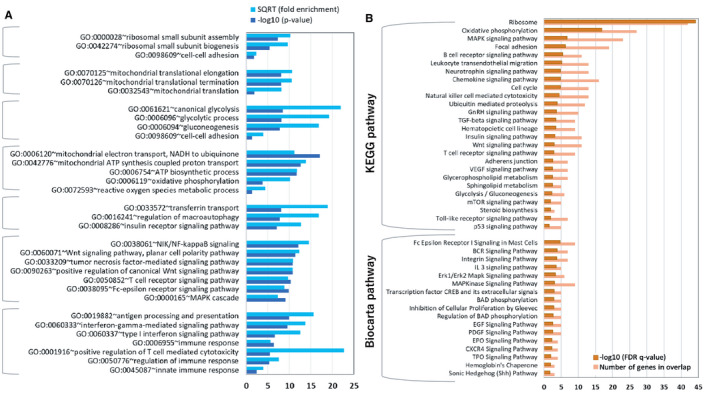
Gene ontology (GO) analysis and canonical pathways between WOL‐treated and control hHSCs. A, Bar graph showing significantly enriched (modified Fisher's exact test, *P* < .05) biological process (BP) GOs between D12 WOL and D12 Control hHSCs. GOs are arranged according to the fold enrichment values of gene functional clustering. A total of seven BP clusters are presented according to the significance of BP in each cluster. SQRT, square root. B, Bar graph showing significantly enriched (FDR *q*‐value) canonical pathways, both KEGG and BioCarta pathways, between D12 WOL and D12 Control hHSCs. GOs and pathways were identified using DAVID online software (https://david.ncifcrf.gov/)

We performed GO cluster analysis to classify highly related genes into functionally related groups using the Gene Functional Classification Tool of DAVID. A total of 29 clusters were identified, setting the classification stringency to medium (default), kappa similarity threshold = 0.35, and multiple linkage threshold to 50%. Figure 4A presents seven clusters of biological functions ranked according to fold enrichment value of each cluster. GOs (BP only) in each cluster are arranged in descending order to their significance (modified Fisher's exact *P*‐value <.05). Top‐ranked BP cluster had enrichment score: 22.1, number of genes in overlap, n = 34, and included BPs of ribosome functions such as ribosomal small subunit assembly (GO:0000028), ribosomal small subunit biogenesis (GO:0042274) and cell‐cell adhesion (GO:0098609). Other important biological events were mitochondrial translation machinery (enrichment score: 11.91; n = 7), glycolytic activity (enrichment score: 10.33; n = 4) and mitochondrial functions such as oxidative phosphorylation and ATP synthesis (enrichment score: 8.5; n = 25). WOL treatment also enriched biological functions of iron homeostasis such as transferrin transport, autophagy and insulin receptor signalling pathway (enrichment score: 7.5; n = 4). Other important biological events in WOL‐treated hHSCs include haematopoiesis‐related signalling pathways such as NIK/NF‐Kappa B signalling, both canonical and non‐canonical Wnt signalling pathways (enrichment score: 7.43; n = 6), and pathways involved in immune responses (enrichment score: 2.73; n = 5).

### 3.8 Significantly enriched canonical pathways between D12 WOL and D12 control hHSCs

To investigate the biological functions of WOL in a network context, we analysed the DEGs between D12 WOL and D12 control cells using the Pathway Viewer feature of the DAVID Functional Annotation Tool. Figure 4B represents the significantly enriched (FDR *q*‐value) canonical pathways (KEGG and BioCarta) arranged in descending order to their significance. Top enriched KEGG pathways included ribosome, oxidative phosphorylation, mitogen‐activated protein kinase (MAPK) signalling pathway, focal adhesion, B cell receptor signalling pathway, leucocyte transendothelial migration, neurotrophin signalling pathway, chemokine signalling pathway and cell cycle. Additionally, haematopoietic cell lineage pathway, gonadotropin‐releasing hormone (GnRH), transforming growth factor‐beta (TGF‐β), Wnt, insulin, T cell receptor, vascular endothelial growth factor (VEGF), mammalian target of rapamycin (mTOR) and Toll‐like receptor signalling pathways were also regulated by WOL treatment. Interestingly, lipid metabolic pathways such as glycerophospholipid metabolism, sphingolipid metabolism and steroid biosynthesis pathways were also enriched in WOL‐treated hHSCs.

Significant BioCarta pathways included Fc epsilon receptor I, B cell receptor (BCR), integrin, interleukin 3 (IL‐3), Erk1/Erk2 MAPK, BCL2‐associated agonist of cell death (BAD) phosphorylation and epidermal growth factor (EGF) signalling pathways. Additionally, erythropoietin (EPO), thrombopoietin (TPO) and Hb's chaperone pathways were also significantly regulated in WOL‐treated hHSCs (Figure 4B).

### 3.9 Gene expressions related to erythroid differentiation in WOL‐treated hHSCs

**FIGURE 5:**
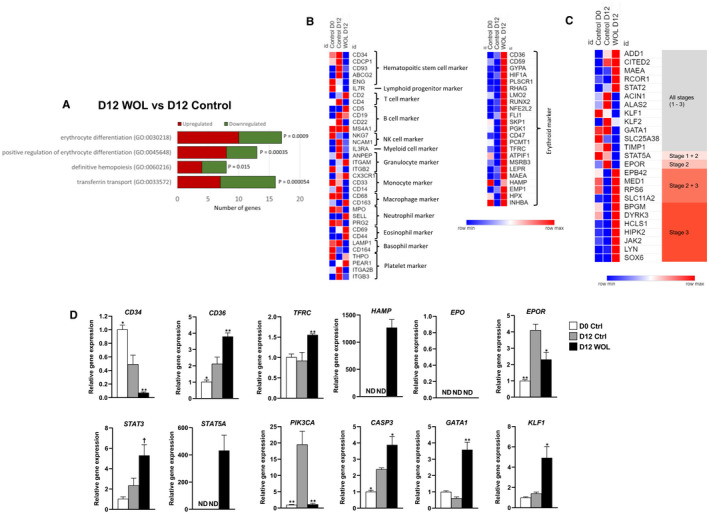
Effect of WOL on differentiation induction in hHSCs. (A) Top significantly enriched biological processes by each set of DEGs are shown. Modified biological processes as per *P*‐value (modified Fisher's exact) by DEGs in D12 WOL vs D12 control was performed using DAVID online tool. Heatmaps by microarray showing relative expression intensity of genes involved in (B) haematopoietic differentiation and (C) erythroid differentiation in hHSCs of D0 control, D12 control and D12 WOL. Heatmaps were generated using an online visualization software Morpheus (https://software.broadinstitute.org/morpheus/). (D) Expressions of genes involved in erythrocyte differentiation in WOL‐treated hHSCs were examined by real‐time PCR. The mRNA expressions were normalized to HPRT1 as internal control and represented as the mean ±SE for n = 4 in a 96‐well PCR plate. Statistically significant difference from the D12 control group (0 µg/mL WOL) at ^†^ *P* < .1, ^*^ *P* < .05 and ^**^ *P* < .01 by Dunnett's multiple comparison test

We found that some specific GOs of erythroid differentiation were significantly regulated by the DEGs between D12 WOL *vs* D12 control such as erythrocyte differentiation (GO:0030218), positive regulation of erythrocyte differentiation (GO:0045648), definitive haemopoiesis (GO:0060216) and transferrin transport (GO:0033572) (Figure 5A). The top 15 up‐regulated genes associated with erythroid differentiation in WOL‐treated hHSCs are listed in Table 2.

**TABLE 2:** Top 15 up‐regulated genes associated with erythroid differentiation in WOL‐treated hHSCs as determined by DNA microarray

Gene symbol	Gene title	Fold change (D12 WOL vs D12 Ctrl)	*P*‐value	Biological process
*ADD3*	Adducin 3 (gamma)	4.56	2.74E‐13	ADD3 composes an adducin which stabilizes interaction between spectrin and actin in erythrocyte.
*LYN*	LYN proto‐oncogene, Src family tyrosine kinase	2.74	1.90E‐11	Activated LYN induces granulopoiesis through HCLS1 phosphorylation.
*SLC11A2*	Solute carrier family 11 member 2	2.44	1.74E‐08	SLC11A2 is transmembrane iron transporter related to cellular iron uptake.
*HIPK2*	Homeodomain interacting protein kinase 2	2.31	1.29E‐07	HIPK2 is involved in haemoglobin biosynthesis and enucleation.
*CASP3*	Caspase 3	2.29	6.18E‐09	CASP3 induce apoptosis, but necessary to prepare the cells for enucleation.
*RCOR1*	REST corepressor 1	2.22	4.09E‐09	RCOR1 drives erythroid differentiation through demethylation of nucleosomal histone H3K4 residues.
*ADAR*	Adenosine deaminase acting on RNA	1.79	1.51E‐09	ADAR converts adenosine residues to inosine in RNA, which is essential for normal erythropoiesis.
*ADD1*	Adducin 1 (alpha)	1.41	5.47E‐07	ADD1 composes an adducin that stabilizes interaction between spectrin and actin in erythrocyte.
*STAT2*	Signal transducer and activator of transcription 2	1.22	.0001	STAT2 is one of the STAT family members, which involved in erythropoiesis via the JAK/STAT pathway.
*MED1*	Mediator complex subunit 1	1.17	.0003	Med1 is a component of the mediator complex, activates GATA‐1‐dependent transcription and regulates erythropoiesis.
*JAK2*	Janus kinase 2	1.09	.0022	JAK2 is one of JAK family members, which is involved in erythropoiesis via the JAK/STAT pathway.
*HCLS1*	Haematopoietic cell‐specific Lyn substrate 1	1.09	.0065	HCLS1 are essential for G‐CSF–triggered granulopoiesis
*BPGM*	Bisphosphoglycerate mutase	1.09	.0082	BPGM regulates Hb affinity for O_2_ through the 2‐3BPG synthesis in the erythrocyte.
*EPB42*	Erythrocyte membrane protein band 4.2	1.08	.019	EPB42 is an ATP‐binding protein that stabilizes the membrane cytoskeleton in erythrocytes.
*ADD2*	Adducin 2 (beta)	1.08	.0077	ADD2 composes an adducin which stabilizes the interaction between spectrin and actin in erythrocyte.

Next, we investigated individual gene expression profile in D0 and D12 controls and D12 WOL‐treated cells. Heatmaps in Figure 5B show relative signal intensities of genes related to haematopoietic and erythrocytic differentiation in hHSCs from microarray analysis. We found that undifferentiated HSC markers such as *CD34*, *CD93*, CUB domain‐containing protein 1 (*CDCP1*), ATP‐binding cassette subfamily G member 2 (*ABCG2*) and endoglin (*ENG*) were consistently down‐regulated in WOL‐treated cells compared to D12 control. The expressions of markers of each haematopoietic lineage—lymphoid progenitor, T cell, B cell, NK cell, myeloid progenitor, granulocyte, monocyte, macrophage, neutrophil, basophil and platelet, were inconsistently regulated, whereas the markers of erythroid lineage such as *CD36*, *CD59*, *GYPA*, hypoxia‐inducible factor 1 alpha (*HIF1A*), *CD47* and transferrin receptor (*TFRC*) were consistently significantly up‐regulated in the WOL‐treated hHSCs. Additionally, several erythroid transcription factors such as LIM domain only 2 (*LMO2*), runt‐related transcription factor 2 (*RUNX2*), nuclear factor, erythroid 2‐like 2 (*NFE2L2*), and Fli‐1 proto‐oncogene, ETS transcription factor (*FLI1*) also showed higher expressions in WOL‐treated cells compared to untreated control cells.

Figure 5C shows the relative signal intensities of erythrocyte differentiation–associated genes according to erythroid maturation stages. For the list of stage‐specific genes, we followed a previously published paper that investigated gene expression profiles of primary human bone marrow–derived nucleated erythroid precursors at three different stages of erythroid maturation.^19^ Stage 1 refers to early maturation stage (proerythroblasts), stage 2 refers to intermediate maturation stage (basophilic erythroblasts) and stage 3 refers to late maturation stage (both polychromatic and orthochromatic erythroblasts).^20^ We found that several erythrocyte differentiation–related genes that are expressed across all three erythroid maturation stages were significantly up‐regulated in WOL‐treated cells, namely adducin 1 (*ADD1*), Cbp/P300 interacting transactivator with Glu/Asp rich C‐terminal domain 2 (*CITED2*), macrophage erythroblast attacher (*MAEA*), REST Corepressor 1 (*RCOR1*) and signal transducer and activator of transcription (STAT) 2 (*STAT2*). On the other hand, some early erythroid differentiation markers such as GATA‐binding factor 1 (*GATA1*), Krüppel‐like factor 1 (*KLF1*), *KLF 2* and *STAT5A* were found down‐regulated in D12 WOL cells in microarray analysis. Erythropoiesis‐related genes that are expressed in later stages of erythroid maturation were found systematically up‐regulated in D12 WOL hHSCs. The late stage–specific genes were solute carrier family 11 member 2 (*SLC11A2*) and homeodomain interacting protein kinase 2 (*HIPK2*), erythrocyte membrane protein band 4.2 (*EPB42*), mediator complex subunit 1 (*MED1*), haematopoietic cell–specific lyn substrate 1 (*HCLS1*) and ribosomal protein S6 (*RPS6*).

Next, we evaluated the expressions of key genes in real‐time PCR (Figure 5D). We found that undifferentiated HSC marker *CD34* was markedly down‐regulated in WOL‐treated cells compared to both D0 and D12 controls, whereas erythroid markers, *CD36* and *TFRC*, were up‐regulated in D12 WOL hHSCs compared to both D0 and D12 control hHSCs. The expressions of *GYPA* and hepcidin (*HAMP*) were detectable only in WOL‐treated cells. Interestingly, the expressions of some master transcription factors in erythropoiesis such as *GATA1*, *KLF1* and *STAT*s were found to be significantly up‐regulated in D12 WOL cells compared to D12 control cells in real‐time PCR tests despite their decreased expression in microarray analysis. The expression of an effector caspase (*CASP3*) was also up‐regulated in treated cells compared to untreated controls. However, *EPO* could not be detectable in all three conditions, whereas the expressions of erythropoietin receptor (*EPOR*) and phosphatidylinositol‐4,5‐bisphosphate 3‐kinase catalytic subunit alpha (*PIK3CA*) were significantly down‐regulated in WOL‐treated cells compared to D12 control cells.

### 3.10 Expression profile of genes related to ‘oxygen homeostasis/response to hypoxia or oxidative stress’, iron homeostasis and haem metabolism in WOL‐treated hHSCs

**FIGURE 6:**
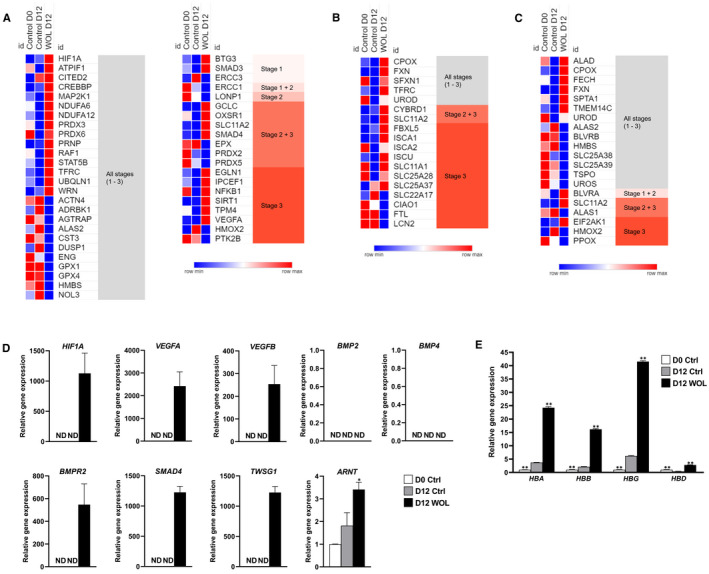
Effect of WOL on expressions of genes involved in oxygen homeostasis/response to hypoxia or oxidative stress, iron homeostasis and haemoglobin synthesis in hHSCs. Heatmaps by microarray showing relative expression intensity of genes involved in (A) oxygen homeostasis/response to hypoxia or oxidative stress, (B) iron homeostasis and (C) haem metabolism in hHSCs of D0 control, D12 control and D12 WOL. Expressions of genes involved in (D) hypoxia‐induced erythropoiesis and iron homeostasis, and (E) haemoglobin subunits in WOL‐treated hHSCs were examined by real‐time PCR. The mRNA expressions were normalized to HPRT1 as internal control and represented as the mean ± SE for n = 4 in a 96‐well PCR plate. Statistically significant difference from the D12 control group (0 µg/mL WOL) at ^†^ *P* < .1, ^*^ *P* < .05 and ^**^ *P* < .01 by Dunnett's multiple comparison test

We investigated expression profile of genes related to important erythroid functions such as oxygen homeostasis (Figure 6A), iron metabolism and homeostasis (Figure 6B), and haem metabolism (Figure 6C) arranged in erythroid maturation stage‐specific manner in the heatmaps. Several oxidative stress–related genes, which are expressed across all three stages of maturation, were up‐regulated in the D12 WOL cells compared to D12 control cells such as hypoxia‐inducible factor 1A (*HIF1A*) and *TFRC*. Stage 1–specific genes B cell translocation gene 3 (*BTG3*) and mothers against decapentaplegic (SMAD) homolog 3 (*SMAD3*) were also up‐regulated in D12 WOL‐treated hHSCs; however, genes that are expressed in later stage 1 and early stage 2 were down‐regulated such as excision repair cross‐complementation (*ERCC*) group 1 (*ERCC1*), *ERCC2* and lon peptidase 1 (*LONP1*). Genes involved in the latest maturation stage oxidative phosphorylation were stably up‐regulated in D12 WOL cells such as solute carrier family 11 member 2 (*SLC11A2*), vascular endothelial growth factor A (*VEGFA*) and *SMAD4*.

Next, we investigated the expression profile of stage‐specific iron homeostasis–related genes (Figure 6B). We found that genes expressed across all three stages (coproporphyrinogen oxidase [*CPOX*] and *SLC11A2*) and at the latest stages of maturation (several solute carrier proteins, and iron‐sulphur cluster assembly protein A‐type [*ISCA*] and U‐type [*ISCU*]) were predominantly up‐regulated in WOL‐treated hHSCs compared to untreated control hHSCs.

Figure 6C shows relative signal intensities of genes related to haem metabolism—the most important function of erythrocytes. We found that the mRNA expressions of aminolevulinate dehydratase (*ALAD*), coproporphyrinogen oxidase (*CPOX*) and ferrochelatase (*FECH*), which encode the 2nd, 6th and 8th enzymes of haem biosynthetic pathway, respectively, were significantly up‐regulated in D12 WOL compared to D12 control cells. Frataxin (*FXN*), one of the components of ISC machinery responsible for the generation of Fe‐S clusters, was also up‐regulated in the treatment cells.

As we found increased expressions of certain genes related to hypoxia‐induced erythropoiesis and iron homeostasis, we further validated their expressions in real‐time PCR (Figure 6D). The expressions of *HIF1A*, *VEGFA*, *VEGFB* and *SMAD4* could be detected only in WOL‐treated cells and were undetected in control cells. However, hypoxia‐induced iron metabolism–related genes bone morphogenetic protein (BMP) 2 (*BMP2*) and *BMP4* could not be detected in both treated and untreated hHSCs. Interestingly, BMP receptor type 2 (*BMPR2*) and twisted gastrulation BMP signalling modulator 1 (*TWSG1*) were detectable only in D12 WOL group but were undetectable in control groups. The expression of aryl hydrocarbon receptor nuclear translocator (*ARNT*) was significantly up‐regulated in WOL‐treated hHSCs compared to D12 control.

Figure 6E shows relative gene expressions of Hb subunits from real‐time PCR experiments. WOL treatment for 12 days significantly increased the expressions of all Hb subunits compared to the untreated D12 control cells. Particularly, mRNA level of *HBG* showed the highest increase in expression followed by *HBA*, *HBB* and *HBD* in descending order in D12 WOL compared to D12 control.

**FIGURE 7:**
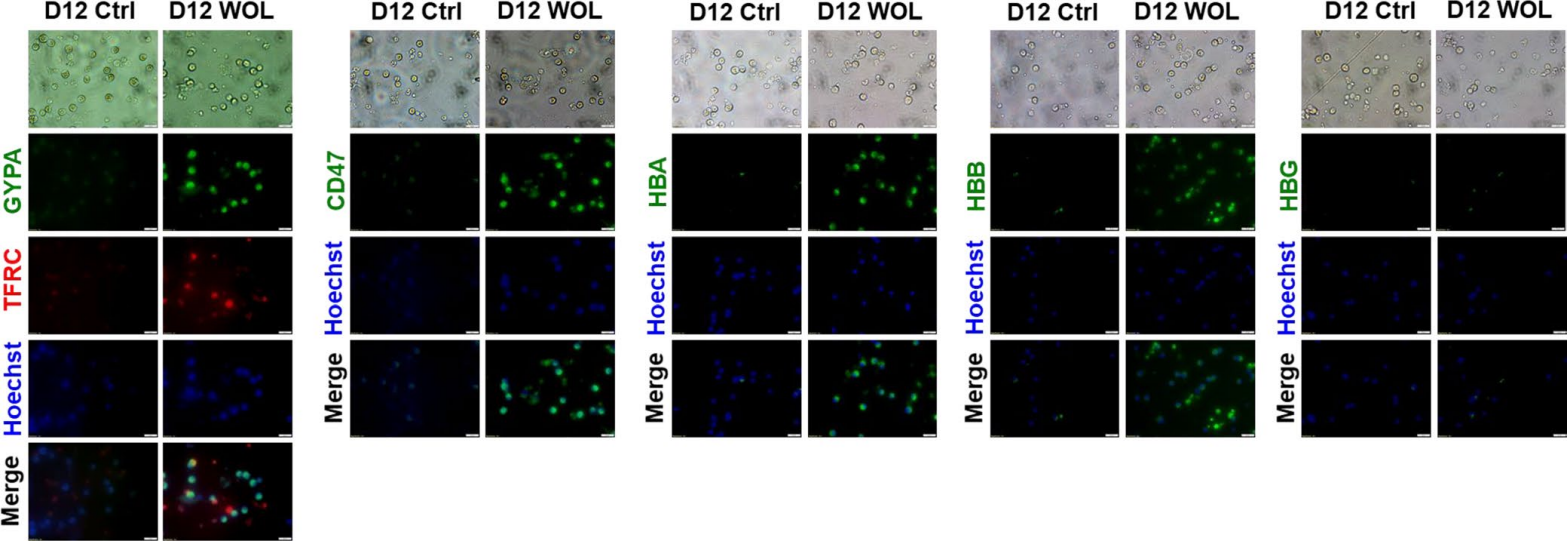
Immunofluorescence staining for erythroid markers and haemoglobin subunits in WOL‐treated hHSCs. The hHSCs were treated with 40 µg/mL WOL for 12 days and then stained using primary antibodies for GYPA, TFRC, CD47, HBA, HBB and HBG. Nuclei in the cells were stained using 1 µg/mL Hoechst 33342. Images were collected with an Olympus IX83 inverted microscope at a magnification of 400×. Scale bars represent 20 μm

**FIGURE 8:**
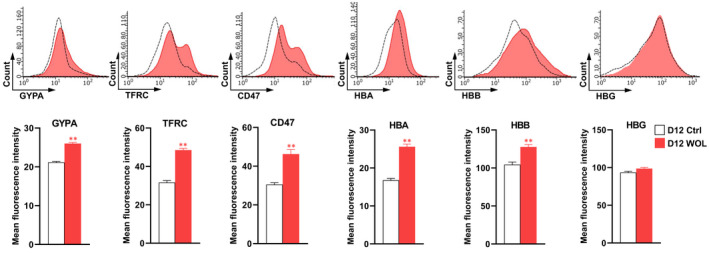
Flow cytometric analysis for erythroid markers and haemoglobin subunits in WOL‐treated hHSCs. The hHSCs were treated with 40 µg/mL WOL for 12 d and then stained using primary antibodies for GYPA, TFRC, CD47, HBA, HBB and HBG. Mean fluorescence intensity (MFI) of the stained cells in histogram were evaluated from acquired 5000 events using a Guava easyCyte™ 8HT Flow Cytometer. The data were represented as the mean ± SE for n = 4. ^**^ *P* < .01 by unpaired two‐tailed Student's *t* test

Figure 7 shows protein expression of erythroid markers and Hb subunits on immunofluorescent staining. WOL treatment for 12 days significantly increased the expressions of erythroid markers (GYPA, TFRC and CD47) and adult Hb subunits (HBA and HBB), but not expression of HBG. Flow cytometric analysis (Figure 8) also shows consistent result with the immunofluorescent staining.

## 4 DISCUSSION

**FIGURE 9:**
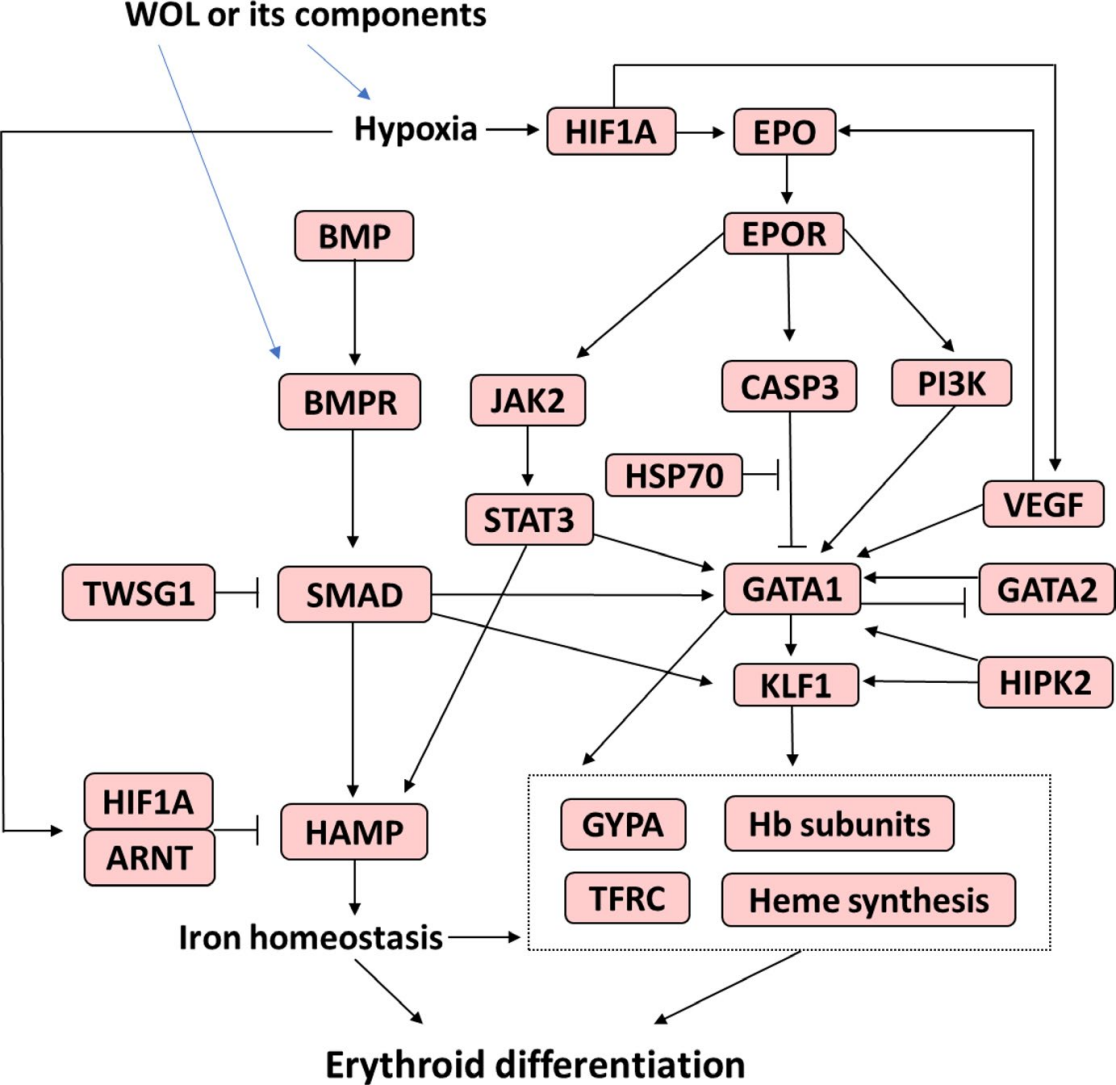
Schematic diagram on events occurring during the erythroid differentiation by WOL

In the present study, we have documented a detailed gene expression profiling of erythropoiesis‐inducing effects of OLE in hHSCs. WOL at a concentration of 40 µg/mL, in the absence of any growth factor, induced erythroid differentiation in hHSCs. Similarly, WOL induced expressions of major erythroid markers and all Hb subunits in a human erythroleukaemic cell line K562 (Figure 9).

We have previously reported hematinic effects of oleuropein‐rich olive leaf tea in a randomized clinical trial.^21^ However, the molecular functions and signalling networks underlying the potential effects of OLE components on HSC fate manipulation and/or on haematological health in human have not been explored. In the present study, HPLC analysis showed oleuropein and hydroxytyrosol were the two most prominent phenolic compounds in the WOL. Although the erythroid differentiation‐inducing effects of these two compounds on hHSCs have not been reported before, our previous study showed that ethanol extract of olive leaf (EOL) containing these compounds could induce differentiation in K562 cells towards monocyte/macrophage lineage.^12^ However, the concentrations of both oleuropein and hydroxytyrosol were much lower in EOL compared to those in the WOL used in this present study. Therefore, findings from our current and previous studies suggest that depending on phenolic constituents, WOL may show different lineage‐specific differentiation potential in different types of cells.

Our gene functional clustering analysis using the DEGs between D12 WOL and D12 control hHSCs (Figure 4A) reveals several major functions of WOL—ribosomal biogenesis and mitochondrial translation machinery, glycolytic process, ATP biosynthesis, iron homeostasis and immune response.

The induction of changes during differentiation requires a complex and coordinated regulation of several gene expression processes such as epigenetic, transcriptional and translational regulation. HSCs, like other adult stem cells, have low translational efficiency, which affects their differentiation ability.^22^, ^23^ Converging evidence establishes that ribosomal biogenesis, which is highly regulated in stem cells to maintain stem cell homeostasis, is also a crucial and constitutive molecular machinery that initiates gene‐specific regulations of translation in stem cells to ensure proper fate decisions.^24^, ^25^, ^26^ A notable recent study shows that global reduction in ribosome levels in Diamond‐Blackfan anaemia, a genetic blood disorder, impairs the translation of major erythroid regulator mRNAs, resulting in a selective reduction in erythroid precursors and progenitors in patients' bone marrows.^27^ In our study, we found that the topmost enriched function of WOL was ribosomal small subunit biogenesis (GO:0042274), followed by mitochondrial translational elongation and termination. The top significant KEGG pathway was ‘Ribosome’. The most significantly enriched hallmark gene set is ‘A subgroup of genes regulated by MYC ‐ version 1’. Myc, a proto‐oncogenic transcription factor, is a well‐known master regulator of ribosome biogenesis.^28^ Additionally, the mTOR pathway that regulates the efficiency of translation through integrating intra‐ and extracellular cues^29^, ^30^ was also significantly up‐regulated. Enriched hallmark gene sets also include ‘genes up‐regulated through activation of mTORC1 complex’ and ‘Genes up‐regulated by activation of the PI3K/AKT/mTOR pathway’. Another major regulator of translation is the mitogen‐activated protein kinase (MEK) pathway, which adjusts translational efficiency to promote cell survival and growth. WOL treatment significantly enriched MAPK cascade (GO:0000165), MAPK signalling pathway, Erk1/Erk2 MAPK signalling pathway and glycolysis pathway. Also, regulation of genes involved in p53 pathways and networks as well as genes involved in protein secretion pathway (hallmark gene sets) corroborates modulation of ribosomal biogenesis^31^, ^32^ in WOL‐treated hHSCs. Most importantly, WOL significantly up‐regulated the mRNA expressions of major erythroid regulators such as *GATA1*, *KLF1*, *STAT3*, *STAT5A*, *LMO2*, *RUNX2*, *NFE2L2* and *FLI1* whose expressions reported to be impaired by reduced ribosome levels.^27^ Based on our findings, it can be reasonably assumed that WOL may influence erythroid fate choice of hHSCs through ribosome‐mediated gene‐specific regulations of translation; thus, it may have implications not only in anaemia but also in other ribosomopathies.

We performed microarray analysis at a single time point, that is on day 12 of WOL treatment when the *GYPA*, an erythroid cell surface marker, was expressed in WOL‐treated hHSCs. Therefore, we further analysed the gene expression profile according to erythrocyte maturation stages.^19^ Firstly, we evaluated the expression profile of all haematopoietic cell lineages (Figure 5B). All the HSC markers were significantly down‐regulated in the D12 WOL cells compared to control hHSCs. Markers of blood cells other than erythroid cells, namely lymphoid progenitor, T cell, B cell, NK cell, myeloid cell, granulocyte, monocyte, macrophage, neutrophil, basophil and platelet, showed inconsistent expressions in WOL‐treated cells. Whereas most of the major erythroid markers and lineage‐specific regulators showed uniform up‐regulation in D12 WOL compared to D12 control cells. The maturation of erythroblasts from CD34+ HSPCs can be assessed by tracking the expression of erythroid lineage markers. HSC‐derived erythroblasts achieve maturation by losing *CD34* and gaining *GYPA* and *TFRC* on their surface. *CD36* is co‐expressed with *GYPA* during the erythroid lineage commitment.^33^, ^34^ In our study, WOL treatment significantly down‐regulated the expression of *CD34*, whereas up‐regulated the expressions of *CD36*, *GYPA* and *TFRC*. On the other hand, the erythroid transcription factor *KLF1* regulates the development of erythroid cells from the bipotent erythroid progenitor cells through the transcriptional activation of erythroid cell–specific gene expression and is responsible for Hb production, regulation of cell cycle and apoptosis. *KLF1* often binds to another major erythroid transcription factor *GATA1* and can act synergistically.^35^, ^36^, ^37^ We found that both *KLF1* and *GATA1* were significantly up‐regulated in WOL‐treated cells compared to controls. Additionally, our protein expression data showed consistent results. Both immunofluorescence staining and flow cytometer analysis showed that WOL treatment for 12 days could significantly up‐regulated the expressions of erythroid markers GYPA, CD47, TFRC and Hb subunits.

We found that WOL treatment regulated biological events related to oxygen homeostasis or response to hypoxia or oxidative stress. For an instance, *HIF1A*, *VEGFA* and *VEGFB* were detected only in WOL‐treated hHSCs. HIF1A is a key mediator of the cellular response to hypoxia that regulates erythropoiesis through EPO.^38^ However, different stimuli such as low iron level, reduced O2 level or increased mitochondrial reactive oxygen species (ROS) have different effects on HIF signalling and lead to activation of different subsets of HIF‐targeted genes.^39^ In our study, WOL did not induce EPO expression despite significant up‐regulation of *HIF1A*. Hypoxic condition also leads to increased *VEGF* expression as the VEGF promoter contains canonical HIF response elements.^40^ However, VEGF can induce erythropoiesis by directly inducing EPO independent of hypoxia condition.^41^ Consequently, EPO and its receptor (EPOR) stimulate the phosphatidylinositol 3‐kinase (PI3K) encoded by *PIK3CA* and STAT via Janus kinase (JAK) for targeting GATA1.^42^, ^43^ We found that WOL treatment down‐regulated the *EPOR* and *PIK3CA* expressions compared to D12 control, whereas up‐regulated the expressions of *STAT3* and *STAT5A*. Although we could not confirm how the expressions of *HIF1A*, *VEGF*s and *EPO* interacted in WOL‐treated hHSCs, our findings suggest that WOL may induce erythropoiesis and regulate iron homeostasis through modulating the expressions of *STAT*s, *GATA1* and *KLF1* but not *PI3K*.

Furthermore, we found that *CASP3* was significantly increased in WOL‐treated hHSCs. *CASP3*, a cysteine protease, plays essential roles in apoptosis but has also been implicated in non‐apoptotic processes like cell differentiation and signalling. It is also involved in erythroid maturation through mitophagy and depletion of enucleation of erythroid cells.^44^, ^45^, ^46^ *CASP3* can arrest erythropoiesis via GATA1 cleavage, but heat shock protein 70 (HSP70) prevents this *CASP3*‐mediated cleavage.^47^ Our findings suggest that WOL treatment may modulate erythroid maturation through CASP3‐mediated mitophagy and enucleation of erythroid cells and subsequently may decrease erythrocyte proliferation and promote cell death of mature cells.

Another important finding of the present study was the expression of *HAMP*, the gene encoding hepcidin, in WOL‐treated hHSCs. HAMP is an iron homeostasis regulator that limits iron export by binding to the iron exporter ferroportin. Disruption of hepcidin level is associated with haematological and renal disorders such as high hepcidin level is associated with chronic kidney disease, whereas decreased hepcidin level is found in iron‐deficiency anaemia.^48^, ^49^ *STAT3* regulates the *HAMP* expression.^50^ *BMP4* and its receptor pathway lead to the *SMAD4* expression, which is a transcriptional activator of HAMP. Also, *BMP4* regulates *KLF1* expression through *GATA1*.^39^, ^51^ On the other hand, *TWSG1* and *ARNT* negatively regulate HAMP expression by inhibiting *SMAD* expression and forming a heterodimer with HIF1A, respectively, thus, maintain iron homeostasis.^39^, ^52^ In real‐time PCR, *BMPR2* and *TWSG* were expressed only in WOL‐treated hHSCs, while *BMP2* and *BMP4* remain undetected in all three groups. *ARNT* was significantly up‐regulated in D12 WOL compared to D12 control cells. Altogether, these findings suggest the possible role of WOL on *BMPR2* in the absence of BMPs and subsequently maintaining iron homeostasis through regulating *HAMP* expression via *SMAD*, *STAT3*, *TWSG1* and *ARNT*. Furthermore, the up‐regulation of *TFRC* by WOL treatment may also play an essential role in iron homeostasis by allowing the cellular uptake of transferrin‐bound iron.^53^ Additionally, we found in microarray analysis that WOL treatment up‐regulated the expressions of several iron homeostasis‐related genes, predominantly, which can be expressed across all three and the latest stages of erythrocyte maturation. Several solute carrier family transporters that are implicated in iron transfer and transport exclusively during the latest erythrocyte maturation stage, such as *SLC11A2*, *SLC25A37* and *SLC25A28*, were up‐regulated in WOL‐treated hHSCs compared to untreated control.^54^, ^55^ Additionally, iron‐sulphur cluster assembly protein A‐type (*ISCA*) and U‐type (*ISCU*), which are involved in iron assembly and homeostasis through biogenesis of iron‐sulphur clusters^56^ at the latest stage of erythrocyte maturation, were also up‐regulated by WOL treatment.

The gene expressions of all Hb subunits, *HBG*, *HBA*, *HBB* and *HBD* (in descending order of their expression values), were up‐regulated by WOL treatment. However, the protein expression analyses on D12 WOL‐treated hHSCs confirmed the significantly increased expression of adult Hb subunits—HBA and HBB. The gamma‐globin subunit is contained in foetal Hb and is highly expressed during the foetal period. In adults, >98% of all Hb is comprised of adult Hb that contains alpha and beta subunits. Thus, gamma‐globin genes are poorly expressed (<1%) in adults. However, increased expression of gamma‐globin leads to higher foetal Hb levels in red blood cells that, in turn, compensate defective β‐globin subunits and, therefore, has some clinical relevance in the treatment of haemoglobinopathies including sickle cell anaemia and beta‐thalassaemia.^57^, ^58^ Additionally, some key regulators of haem biosynthesis pathway such as *ALAD*, *CPOX* and *FECH* ^59^ were up‐regulated by WOL treatment.

Another important function observed in WOL‐treated cells was regulation of several interconnected metabolic pathways. Significantly enriched hallmark gene set included ‘Genes encoding proteins involved in metabolism of fatty acids’, ‘genes involved in cholesterol homeostasis’ and ‘genes encoding proteins involved in glycolysis’. Significantly enriched KEGG pathways include ‘glycerophospholipid metabolism’, ‘sphingolipid metabolism’, ‘glycolysis’ and ‘steroid biosynthesis’. Previously it was reported that interconnections between the distinct metabolic pathways are unique for each lineage during HSC differentiation.^60^ Future metabolomics study would clarify the metabolic and transcriptional changes involved in fate choice influenced by WOL in hHSCs.

Altogether, our study presents an in‐depth transcriptional analysis of biological functions induced by WOL in hHSCs and highlights its multilevel exploitable targets for treating and/or preventing anaemia and other haematological disorders. However, further integrative studies at the proteome and metabolome levels and their validation in in vivo settings are required to confirm our findings.

## CONFLICT OF INTEREST

KY, SS and MY are employed by Nutrition Act Co., Ltd. The remaining authors declare that the research was conducted in the absence of any commercial or financial relationships that could be construed as a potential conflict of interest.

## AUTHOR CONTRIBUTIONS

**Shinji Kondo:** Data curation (equal); Formal analysis (equal); Investigation (equal); Validation (equal); Visualization (equal); Writing‐original draft (equal). **Farhana Ferdousi:** Data curation (equal); Formal analysis (equal); Investigation (equal); Validation (equal); Visualization (equal); Writing‐original draft (equal). **Ken Yamauchi:** Resources (lead). **Sofya Suidasari:** Resources (supporting). **Miki Yokozawa:** Resources (supporting). **Mohamed Moncef Harrabi:** Resources (lead). **Ken‐ichi Tominaga:** Data curation (supporting); Formal analysis (supporting); Investigation (supporting). **Hiroko Isoda:** Conceptualization (lead); Funding acquisition (lead); Project administration (lead); Supervision (lead); Writing‐review & editing (lead).

## Supporting information

App S1

## Data Availability

Microarray data are deposited in the Gene Expression Omnibus (GEO) under accession number GSE148775 (https://www.ncbi.nlm.nih.gov/geo/query/acc.cgi?acc=GSE148775).
