# Supplementary material for: Comprehensive transcriptome analysis of erythroid differentiation potential of olive leaf in haematopoietic stem cells
Source: J Cell Mol Med. 2021 Jun 27;25(15):7229–43. doi: 10.1111/jcmm.16752 (PMC8335692; doi:10.1111/jcmm.16752)
Supplement: Supplementary file 1 — App S1 [file JCMM-25-7229-s001.docx]

**
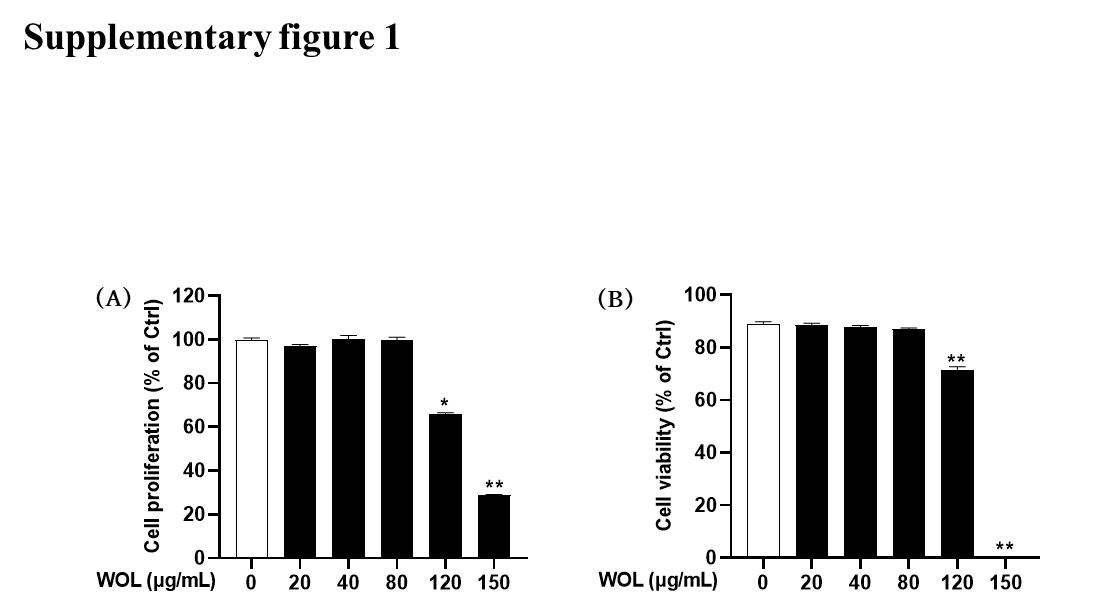
**

**Supplementary Figure 1: Effects of WOL on proliferation and viability of K562 cells**

The K562 cells were treated with 0 - 150 µg/mL WOL for 6 days. (**A**) The proliferation of WOL-treated cells was assessed by MTT assay. (**B**) The viability of WOL-treated cells was measured by flow cytometry. Each value is expressed as % of control (0 µg/mL WOL) in proliferation and % of total counted cells in viability, and then represent the mean ± SE for n = 4 in a 96-well plate. Statistically significant difference from the control group at ^*^*P* < 0.05 and ^**^*P* < 0.01 by Dunnett’s multiple comparison tests.


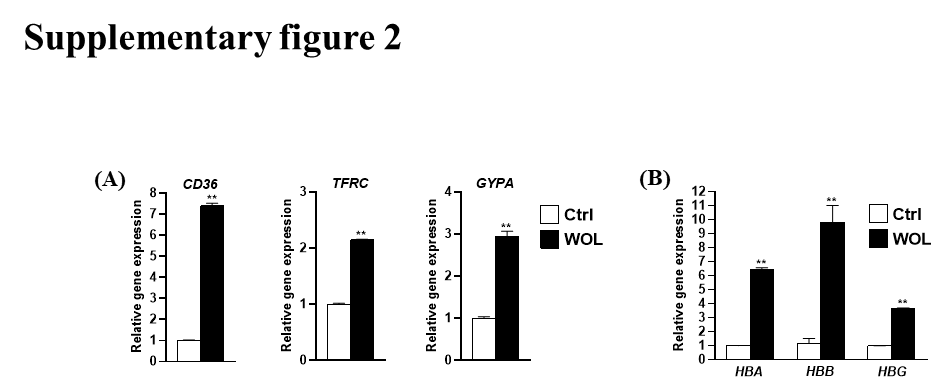


**Supplementary Figure 2:** **Effect of WOL on the gene expressions of erythroid markers and** **hemoglobin subunits in K562 cells**

The K562 cells were treated with 120 µg/mL WOL for 6 days. Gene expressions of erythroid markers (**A**) and hemoglobin subunits (**B**) in WOL-treated cells were examined by real-time PCR. The mRNA expressions were normalized to β-actin as internal control and represent the mean ± SE for n = 4 in a 96-well PCR plate. Statistically significant difference from the control group (0 µg/mL WOL) at ^**^*P* < 0.01 by two-tailed Student’s t-test.


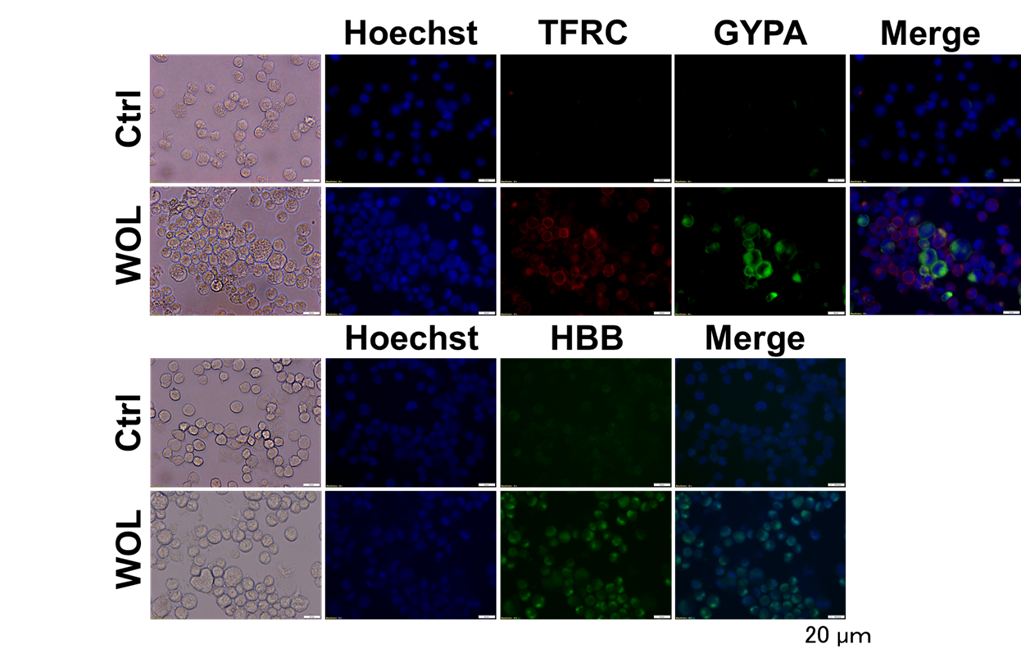


**Supplementary Figure 3: Immunofluorescence staining for erythroid markers and hemoglobin in WOL-treated K562 cells**

The K562 cells were treated with 120 µg/mL WOL for 6 days and then stained using primary antibodies for TFRC, GYPA, and Hbβ. Nuclei in the cells were stained using 1 µg/mL Hoechst 33342. Images were collected with an Olympus IX83 inverted microscope at a magnification of 400×. Scale bars represent 20 μm

**Supplementary Materials and Methods**

**Extraction of olive leaves**

Fresh olive leaves were harvested from a farm in Siliana province at the central west of Tunisia in the middle of December 2018 and were shed-dried at the room temperature (RT) between 20-25℃. The dried leaves were powdered with a food processor. Dried leaves powder (25 g) was steeped in 500 ml of sterile distilled hot water at 90℃ for 30 min with a hot bath and then was cooled down at RT. The decoction solution was filtered with stericup and steritop vacuum driven sterile filters (hole diameter 0.22µm, SCGPT05RE, Merck, Darmstadt, Germany) and evaporated with a rotary evaporator to half volume. The water extract was protected from light with aluminum foil and kept at －80℃ in a deep freezer.

**HPLC analysis**

Filtered OLE (50 mg/mL) was injected (10 μL) into an HPLC system for quantitative analysis (SHIMAZDU CORP, Kyoto). The elution conditions were as follows: mobile phase A (10% Formic acid in water) and mobile phase B (ACN:MeOH = 1:1), the flow rate of 1 mL/min, and operating temperature 40°C. The running gradient was 0 - 100%, 40 min. Re-equilibration duration was 10 min. The wavelength was set at 280 nm for monitoring Apigenin, Luteolin, Hydroxytyrosol, and Oleuropein, and at 331 nm for monitoring Apigenin-7-O-glucoside and Luteolin-7-O-glucoside.

**Cell culture and sample treatment**

The human chronic leukemia cell line K562 was obtained from the Riken Cell Bank (RCB0027, Tsukuba, Ibaraki, Japan). The cells were maintained in RPMI 1640 medium (Gibco Waltham, MA, USA), supplemented with 10% heat-inactivated fetal bovine serum (FBS; 10270-106, Gibco Waltham, MA, USA) and 1% Penicillin/Streptomycin solution (LONZA, Basel, Switzerland) in 75 cm^2^ cell culture flask. The cultured flask was incubated at 37°C in a humidified incubator with 5% CO_2_ and passaged every 2 days. The cells were seeded at a concentration of 2 × 10^4^ cells/ml in the supplemented RPMI 1640 medium and on the following day treated with WOL for a total of 6 days. The WOL-containing medium was renewed at 3 days after starting treatment. Human bone marrow CD34＋ progenitor cells were purchased from Lonza Inc. (Lonza, Basel, Switzerland). The cells were maintained in StemPro^Ⓡ^-34 complete medium containing StemPro^Ⓡ^-34 SFM medium (Gibco, Waltham, MA, USA), StemPro^Ⓡ^-34 nutrient supplement, 100 ng/mL recombinant human stem cell factor (SCF; PHC2115, Gibco, Waltham, MA, USA), 50 ng/mL recombinant human interleukin-3 (IL-3; PHC0034, Gibco, Waltham, MA, USA) and 25 ng/mL recombinant human granulocyte-macrophage colony-stimulating factor (GM-CSF; PHC2015, Gibco, Waltham, MA, USA) at 37 ℃ in humidified 95％ with 5% CO^2^ for 7 days (day 0 – day 7) on 60 mm dish. On day 8, the cells were seeded at a concentration of 2 × 10^4^ cells/ml in the complete medium in a 24-well plate. From day 9 to day 21 (D0 to D12), the cells were treated with or without 40 µg/mL WOL. The medium was changed every 3 days.

**Cell proliferation assay**

K562 cell-containing medium were transferred to a 96 well-plate (30 µl) and then 70 μl of MTT solution was added into each well. After 24 h, 100 μl of 10% SDS solution was added into each well. The optical density was measured at 570 nm using a Varioskan Lux multimode microplate reader (Thermo Fisher, Waltham, MA, USA).

**Cell viability assay**

K562 cell-containing medium was transferred to a round-bottom 96 well-plate (20 µl) and suspended by 180 µl Guava ViaCount reagent (Luminex Corporation, Austin, TX, USA). The cell solution was incubated for 5 min. The viable cells were counted with a Guava EasyCyte™ 8HT Flow Cytometer (Luminex Corporation, Austin, TX, USA).

**Colony formation assay**

5×10^4^ cells after treatment for 12 days were suspended in Human Methylcellulose Serum-Free Enriched Media (HSC005SF, R&D Systems, Minneapolis, MN, USA), and plated in 35-mm culture dishes. The cells in dishes were incubated for 14 days at 37 ℃ in humidified 95％ with 5% CO^2^. Colonies in the dishes were counted using an inverted microscope and classified as colony-forming unit-erythroid (CFU-E), burst-forming unit-erythroid (BFU-E), colony-forming unit-granulocyte macrophage (CFU-GM), and colony-forming unit-granulocyte erythroid macrophage megakaryocyte (CFU-GEMM) according to their morphology.

**Immunofluorescence analysis**

For immunofluorescent staining proteins after treatment, 2.0 × 10^7^ cells of HSCs and K562 cells were plated on the bottom surface of wells in 24-well plate and then air-dried to attach the surface. Cells were fixed with 4% paraformaldehyde for 10 minutes at room temperature. For detecting Hb subunits, cells were permeabilized with 0.1% Triton X‐100 for 10 minutes at room temperature and washed 3 times with PBS. Cells were blocked with 5% goat serum for 1 h at room temperature. All primary antibodies: anti-GYPA antibody (ab129024), anti-TFRC antibody (ab1086), anti-CD47 antibody (ab175388), anti-HBA antibody (ab92492), anti-HBB antibody (ab214049), and anti-HBG antibody, were purchased from Abcam (Cambridge, UK). Blocked cells were incubated with each primary antibody diluted 1:1000 in 1% goat serum overnight at 4℃ and washed three times with PBS. Alexa secondary antibodies: goat anti-rabbit IgG Alexa Fluor 488 (A32731) and goat anti-mouse IgG Alexa Fluor 594 (ab150116) were purchased from Thermo Fisher Scientific (Waltham, MA, USA) and Abcam (Cambridge, UK), respectively. Continuously, cells were incubated with each secondary antibody diluted 1:1000 in 1% goat serum for 1 h at RT and washed three times with PBS. The nuclei were stained with 1 µg/mL Hoechst 33342 (H21492, Thermo Fisher Scientific, Waltham, MA, USA) for 10 min at RT and washed three times with PBS. The stained cells were visualized under an Olympus IX83 inverted microscope (Olympus Life Science, Shinjuku-ku, Tokyo, Japan) using the cellSens imaging software (Olympus Life Science, Shinjuku-ku, Tokyo, Japan).

**Frow cytometry**

For flow cytometric analysis after treatment, HSCs were fixed with 4% paraformaldehyde for 10 minutes at room temperature. For detecting Hb subunits, cells were permeabilized with 0.1% Triton X‐100 for 10 minutes at room temperature. The cells were incubated with each primary antibody diluted 1:1000 in 1% goat serum for 1 hour at room temperature. Continuously, cells were incubated with each secondary antibody diluted 1:1000 in 1% goat serum for 1 hour at room temperature and washed with PBS. Mean fluorescence intensity (MFI) of the stained cells were evaluated from acquired 5000 events using a Guava easyCyte™ 8HT Flow Cytometer (Luminex Corporation, Austin, TX, USA).

**RNA extraction**

Isogen reagent (311-02501, Nippon Gene, Tokyo, Japan) was used to extract total RNA from K562 cells after treatment for 6 days, and from hHSCs on day 9 (D0 before treatment) and day 21 (D12 after treatment), following the manufacturer’s instructions. Concentrations of total RNA were quantified with NanoDrop 2000 spectrophotometer (Thermo Scientific, Wilmington, DE, USA).

**DNA microarray analysis**

DNA microarray was performed on GeneAtlas™ System using GeneChip™ 3' IVT PLUS Reagent Kit and GeneAtlas™ Hybridization, Wash, and Stain Kit for 3’ IVT Arrays (Applied Biosystems, Thermo Fisher Scientific Inc.) following the manufacturer’s instructions. Briefly, biotin-labeled cRNAs were synthesized from 250 ng of total RNA samples following 16 h incubation. The purified and fragmented cRNA (9.4 µg) samples were then hybridized on to Human Genome U219 array strips (HG-U219) for 16 h at 45°C. The array strips were washed and stained in the GeneAtlas Fluidics Station 400 (Affymetrix) and the resulting images were scanned using the GeneAtlas Imaging Station (Affymetrix). The HG-U219 Array Strips are designed with only Perfect Match probes and contain over 530,000 probes covering about 36,000 transcripts and variants, which in turn, represent more than 20,000 unique genes. Gene-level normalization and signal summarization was performed on Expression Console™ Software using Robust Multi-array Analysis (RMA) algorithm. Subsequent analysis of the gene expression data was carried out using Affymetrix® Transcriptome Analysis Console (TAC) ver. 4.0 Software.

Gene annotation, and pathway analysis were conducted using an online data mining tool DAVID ver. 6.8 and the Molecular Signatures Database (MSigDB) ver. 7.1 of the **Gene Set Enrichment Analysis (**GSEA) software. Heat maps were generated using visualization software Morpheus (https://software. broadinstitute.org/morpheus).

**Quantitative** **real-time PCR**

Reverse transcriptions were performed at 20 µl reaction mixes in PCR tube using 4µl SuperScript Ⅳ VILO Master Mix (Invitrogen, Carlsbad, CA, USA) and 16 µl total RNA solution (final RNA concentration: 10 ng/20 µl). The reactions were performed with a thermal cycler MiniAmp Plus (A37835, Applied Biosystems, Foster City, CA, USA) under the following programs: 25 °C for 10 min, followed by 50 °C for 10 min, and 85 °C for 5 min. And then, the synthesized cDNA samples were cooled to 4 °C and stored at −20 °C until performing the real-time PCR. Quantitative real-time PCR analysis were performed to analyze the gene expressions using following primer sets and TaqMan probes: *CD34* (Hs02576480_m1), *CD36* (Hs00354519_m1), *TFRC* (Hs00951083_m1), *GYPA* (Hs01068079_s1), *HBA* (Hs00361191_g1), *HBB* (Hs00758889_s1), *HBG* (Hs00361131_g1), *HBD* (Hs00426283_m1), *HIF1A* (Hs00153153_m1), *EPO* (Hs01071097_m1), *EPOR* (Hs00959427_m1), *STAT3* (Hs00374280_m1), *STAT5A* (Hs00559637_g1), *PIK3CA* (Hs00907957_m1), *CASP3* (Hs00234387_m1), *GATA1* (Hs01085823_m1), *KLF1* (Hs00610592_m1), *VEGFA* (Hs00900055_m1), *VEGFB* (Hs00173634_m1), *BMP2* (Hs00154192_m1), *BMP4* (Hs03676628_s1), *BMPR2* (Hs00176148_m1), *SMAD4* (Hs00929647_m1), *TWSG1* (Hs00221028_m1), *ARNT* (Hs01121918_m1) and *HAMP* (Hs00221783_m1). *ACTB* (Hs03023880_g1) and *HPRT1* (Hs02800695_m1) served as an internal control was used for the normalization of each gene in K562 cells and hHSCs, respectively. TaqMan real-time PCR amplification reactions were performed at 20 μL on a 96-well PCR plate using 10 μL TaqMan Gene Expression Master Mix, (4369016, Applied Biosystems, Foster City, CA, USA), 1 μL each primer/probe mix, and 9 μL template cDNA (final cDNA concentration 200 ng/20 μL). To quantify the mRNA, the amplification reactions were carried out using an AB 7500 Fast Real-Time PCR system (Applied Biosystems, Foster City, CA, USA) under the following thermal cycling programs: 2 min at 50˚C, 10 min at 95˚C, and 50 cycles at 95˚C for 15 seconds followed by 60˚C for 1 min. All reactions were run in tetraplicates and the relative gene expression values were calculated using the 2^-ΔΔCt^ method. If the samples on several reactions with primer/probe mixes could not be amplified by 50 cycles, the genes in the samples were regarded as not detected (ND). In those cases, the Ct values of the ND samples were assumed at 50 for calculating relative gene expressions in WOL-treated cells compared to untreated cells.

**Statistical analysis**

Statistical analyses were performed with GraphPad Prism version 8.0 (GraphPad Software, Inc., San Diego, CA). Data were represented as the mean ± standard error of the mean (SEM). An unpaired two-tailed Student’s t-test was used to compare between two groups. A one-way ANOVA followed by Dunnett’s posthoc test was performed to compare the treatment groups to a control group. A *P*-value < 0.05 was considered as significant.
